# Autoantibody profiling identifies predictive biomarkers of response to anti-PD1 therapy in cancer patients

**DOI:** 10.7150/thno.45816

**Published:** 2020-05-16

**Authors:** Qiaoyun Tan, Dan Wang, Jianliang Yang, Puyuan Xing, Sheng Yang, Yang Li, Yan Qin, Xiaohui He, Yutao Liu, Shengyu Zhou, Hu Duan, Te Liang, Haoyu Wang, Yanrong Wang, Shiyu Jiang, Fengyi Zhao, Qiaofeng Zhong, Yu Zhou, Shasha Wang, Jiayu Dai, Jiarui Yao, Di Wu, Zhishang Zhang, Yan Sun, Xiaohong Han, Xiaobo Yu, Yuankai Shi

**Affiliations:** 1 Department of Medical Oncology, National Cancer Center/National Clinical Research Center for Cancer/Cancer Hospital, Chinese Academy of Medical Sciences & Peking Union Medical College, Beijing Key Laboratory of Clinical Study on Anticancer Molecular Targeted Drugs, Beijing 100021, China.; 2 State Key Laboratory of Proteomics, Beijing Proteome Research Center, National Center for Protein Sciences-Beijing (PHOENIX Center), Beijing Institute of Lifeomics, Beijing 102206, China.; 3 Clinical Pharmacology Research Center, Peking Union Medical College Hospital, Chinese Academy of Medical Sciences & Peking Union Medical College, Beijing Key Laboratory of Clinical PK & PD Investigation for Innovative Drugs, Beijing 100032, China.

**Keywords:** Anti-PD1 therapy, Autoantibody, Biomarker, Protein microarray

## Abstract

**Background**: Programmed cell death protein 1 (PD1) inhibitors have revolutionized cancer therapy, yet many patients fail to respond. Thus, the identification of accurate predictive biomarkers of therapy response will improve the clinical benefit of anti-PD1 therapy.

**Method**: We assessed the baseline serological autoantibody (AAb) profile against ~2300 proteins in 10 samples and ~4600 proteins in 35 samples with alveolar soft part sarcoma (ASPS), non-small-cell lung cancer (NSCLC) and lymphoma using Nucleic Acid Programmable Protein Arrays (NAPPA). 23 selected potential AAb biomarkers were verified using simple, affordable and rapid enzyme linked immune sorbent assay (ELISA) technology with baseline plasma samples from 12 ASPS, 16 NSCLC and 46 lymphoma patients. SIX2 and EIF4E2 AAbs were further validated in independent cohorts of 17 NSCLC and 43 lymphoma patients, respectively, using ELISA. The IgG subtypes in response to therapy were also investigated.

**Results**: Distinct AAb profiles between ASPS, NSCLC and lymphoma were observed. In ASPS, the production of P53 and PD1 AAbs were significantly increased in non-responders (p=0.037). In NSCLC, the SIX2 AAb was predictive of response with area under the curve (AUC) of 0.87, 0.85 and 0.90 at 3 months, 4.5 months, 6 months evaluation time points, respectively. In the validation cohort, the SIX2 AAb was consistently up-regulated in non-responders (p=0.024). For lymphoma, the EIF4E2 AAb correlated with a favorable response with AUCs of 0.68, 0.70, and 0.70 at 3 months, 4.5 months, and 6 months, respectively. In the validation cohort, the AUCs were 0.74, 0.75 and 0.66 at 3 months, 4.5 months, and 6 months, respectively. The PD1 and PD-L1 IgG2 AAbs were highly produced in ~20% of lymphoma responders. Furthermore, bioinformatics analysis revealed antigen functions of these AAb biomarkers.

**Conclusion**: This study provides the first evidence that AAb biomarkers selected using high-throughput protein microarrays can predict anti-PD1 therapeutic response and guide anti-PD1 therapy.

## Introduction

Immune-checkpoint blockade (ICB) has demonstrated to be a powerful cancer treatment option with remarkable clinical response durability 1, especially inhibitors targeting programmed cell death protein 1 (PD1). However, a large portion of cancer patients fail to respond to anti-PD1 therapy 2. Significant efforts have been made to find biomarkers that guide ICB therapy, such as programmed death-ligand-1 (PD-L1) expression, microsatellite instability (MSI)/mismatch-repair deficiency (dMMR), and tumor mutation burden 3-7.

Autoantibodies (AAbs), or antibodies that recognize self-proteins (autoantigens; AAgs), are also of particular interest to ICB research since AAbs can be generated by altered protein expression, mutation (neoantigen), degradation of tumor proteins, and defects in immune tolerance or inflammation 8-10. AAbs also play important roles in the maintenance of host homeostasis by clearing dead cells and distinguishing diseased cells from normal cells 11, 12. PD-L1, which is central to immune checkpoints, can be expressed on tumor cells or B cells and its inhibition can result in the activation and proliferation of T cells 13. The accumulated evidence suggests that immune-related adverse events are associated with AAbs 14-18. For example, 23 AAbs were measured in 133 melanoma patients before and after ipilimumab treatment. The results indicated that patients with anti-thyroid antibodies have more thyroid dysfunction in subsequent anti-PD1 therapy. In addition, patients with AAbs displayed a higher probability of long-term survival and treatment response 14. These results were confirmed by measuring pre-existing AAbs to rheumatoid factor, antinuclear antibody, antithyroglobulin, and antithyroid peroxidase in 137 non-small-cell lung cancer (NSCLC) patients 16.

Among the previously-identified biomarkers for anti-PD1 therapeutic response, PD-L1 immunohistochemistry (IHC) was first approved by the United States Food and Drug Administration (FDA) as a companion test in NSCLC 3. However, the clinical utility of the PD-L1 IHC assay is limited. The sensitivity and specificity of PD-L1 antibodies are inconsistent. Moreover, the clinical thresholds differ across different cancers 3. The predictive value of previously-identified biomarkers of anti-PD1 response is also limited, in part, to the complexity of genomic aberration, transcriptional control, mRNA stability, oncogenic signaling and protein stability in cancer 3. Notably, the combination of different biomarkers may circumvent the limitation of using a single biomarker and improve the efficacy of anti-PD1 therapy 3. Therefore, it is important to identify new biomarkers that can predict the response of anti-PD1 therapy while considering assay accuracy, accessibility, easy-to-use and economic cost.

In this study, we set out to identify circulating AAb biomarkers predictive of anti-PD1 therapeutic response prior to treatment 19, 20. Plasma samples from alveolar soft part sarcoma (ASPS), NSCLC or lymphoma patients prior to anti-PD1 therapy were screened using a protein array platform called Nucleic Acid Programmable Protein Array (NAPPA), which has been widely applied in the identification of AAb biomarkers for cancers and autoimmune diseases 21. Briefly, NAPPA arrays print complementary DNA (cDNA) plasmids encoding for the proteins-of-interest and a fusion tag (i.e., Glutathione S-transferase; GST) onto an aminosilane-modified glass slide 22, 23. At the time of the experiment, the plasmids are transcribed and translated using a human cell-free expression system and captured to the slide *in situ* through pre-immobilized anti-tag capture antibodies. Patient plasma were screened with NAPPA arrays displaying ~6,900 human proteins, which identified 21 highly produced AAb biomarkers targeting antigens associated with cancer. These AAbs, along with PD1 and PD-L1 AAbs, were then verified using enzyme-linked immune sorbent assay (ELISA), a simple, cost-effective, and rapid assay that can be performed easily in the healthcare setting. Moreover, the relationship between the 23-marker AAb panel and the response of cancer patients to the anti-PD1 therapy was investigated.

## Materials and Methods

### Study design and patient characteristics

Plasma samples from patients receiving anti-PD1 therapy (Sintilimab, Toripalimab, Nivolumab) were collected within a week prior to the first treatment between August 2016 and September 2019 from different hospitals. We used same standard operating procedure for all samples. After preparing the plasma using EDTA, the samples were centrifuged twice at 16,000 g and 4°C for 10 minutes, the supernatant was transferred to a new tube. Samples were transported by cold chain and stored at -80°C until use. The freeze-thaw cycles were the same.

There are 4 cohorts of patient sample in this study, the discovery cohort 1 compromised of 4 NSCLC, 3 ASPS and 3 lymphoma patients, the discovery cohort 2 included 13 NSCLC, 12 ASPS and 10 lymphoma patients, there were 16 NSCLC, 12 ASPS and 46 lymphoma patients in verification cohort, the validation cohort consisted of 17 NSCLC and 43 lymphoma patients. The patient baseline characteristics are shown in Table 1. The treatment efficacy was defined as complete response (CR), partial response (PR), stable disease (SD), or progressive disease (PD) according to Response Evaluation Criteria in Solid Tumours (RECIST) version 1.1 for ASPS, NSCLC and International Working Group 2007 Criteria for lymphoma 24. During anti-PD1 therapy, some patients showed response (CR/PR) at the initial stage and later became PD with subsequent treatments. To systematically evaluate the performance of biomarkers to discriminate between responders and non-responders, all patients were evaluated at 3 months, 4.5 months and 6 months 4-6, 25, 26. A “responder” was defined as a patient who had a CR/PR/SD prior to the evaluation time point. A “non-responder” was a patient who had PD on/before the evaluation time point 6, 26.

**Figure 1:**
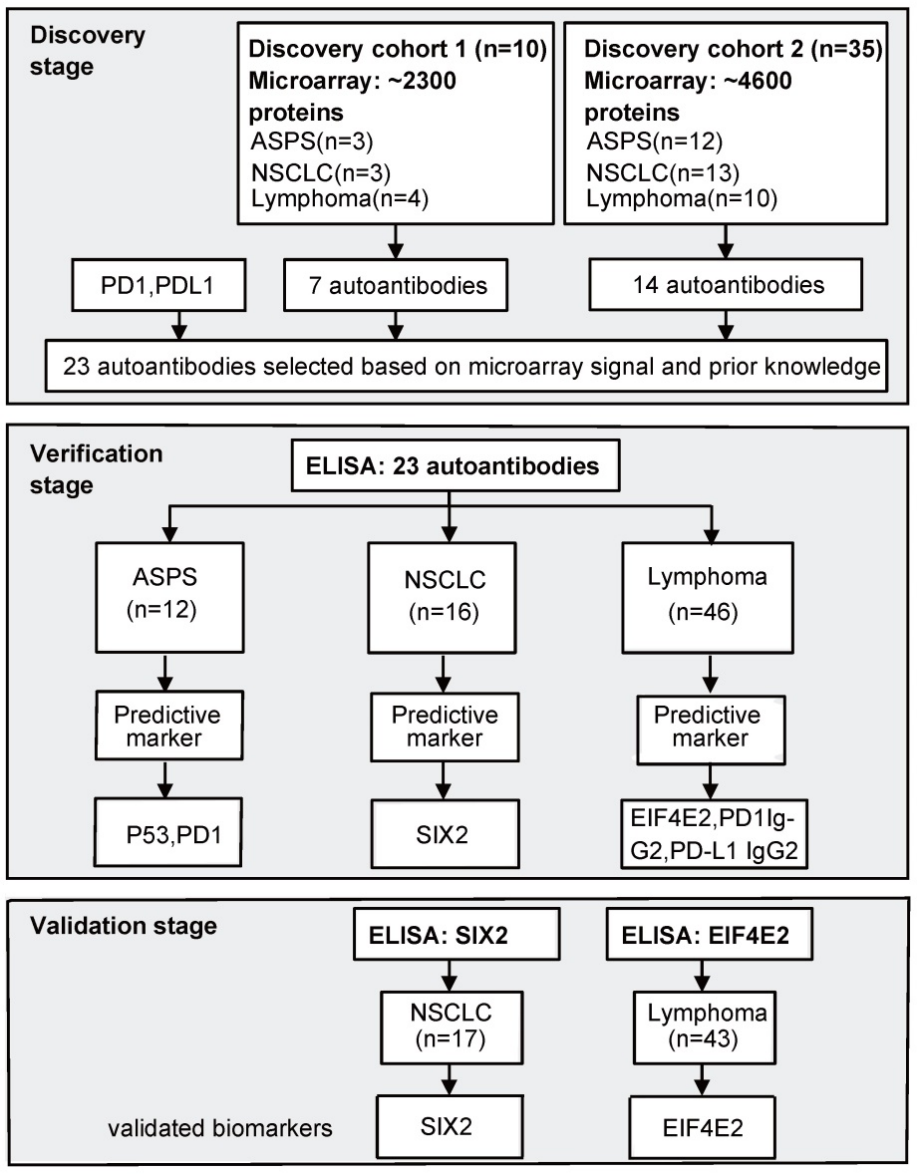
** Study flow chart.** In the discovery stage, we screened AAbs in two sets of plasma samples using NAPPA protein microarrays displaying a total of ~2,300 or ~4,600 human proteins. In the verification stage, selected AAbs based on NAPPA data and prior knowledge were measured using ELISA technology with the baseline plasma samples from ASPS (n=12), NSCLC (n=16) and lymphoma (n=46) patients receiving anti-PD1 therapy. Statistically-significant predictive biomarkers, SIX2 and EIF4E2, were further validated during the validation stage using an independent group of 17 NSCLC and 43 lymphoma patients. ASPS: alveolar soft part sarcoma, NSCLC: non-small-cell lung cancer.

The study was conducted in three stages. In the first stage, NAPPA arrays were consecutively employed to screen AAb profiles against ~ 2300 proteins in discovery cohort 1 (10 samples) and ~ 4600 proteins in discovery cohort 2 (35 samples). Nine of the 10 patient samples in discovery cohort 1 were also used in discovery cohort 2. In the second stage, ELISA technology was used to verify the production level of selected AAbs in 12 ASPS, 16 NSCLC and 46 lymphoma patients (42 Hodgkin's lymphoma [HL], 4 diffuse large B-cell lymphoma [DLBCL]). Finally, AAb biomarkers were validated using an independent cohort of 17 NSCLC and 43 HL patients using ELISA (Figure 1).

All experiments were executed according to the Declaration of Helsinki.

### Identification of plasma AAbs in patients receiving anti-PD1 therapy using protein microarrays

The protein microarray preparation and AAb detection workflow are shown in Figure S1A. The printed NAPPA microarrays were obtained from the NAPPA Protein Array Core (Arizona State University; Tempe, Arizona, USA). The ~2300 proteins screened with the discovery cohort 1 belong to numerous protein classes, including nucleic acid binding proteins, translational proteins, protein modifying enzymes, protein-binding modifying enzymes and metabolite interconversion enzymes (Figure S1B). The ~4600 proteins screened with the discovery cohort 2 mainly consist of transcriptional regulators, protein-binding activity modulators, protein modify enzymes, nucleic acid binding and scaffold/adaptor proteins (Figure S1C).

To perform plasma AAb screening, the protein microarray was first blocked with 5% milk in PBST (PBS, 0.2% Tween) for 1 hour, and incubated with plasma diluted at 1:50 in 5% milk for 16 hours at 4 °C. After washing the slide three times with PBST, 1:500 diluted Alexa Fluor 647 goat anti-human IgG was added to bind to the human AAbs. Fluorescence signal was detected and analyzed using the GenePix 4300A microarray scanner (Molecular Devices, Sunnyvale, CA, USA) and GenePix Pro7 software (Molecular Devices, Sunnyvale, CA, USA), respectively. The reactivity of each AAb was quantified by the signal intensity of spots with a “Halo ring” according to the method described previously 27.

Briefly, during *in vitro* transcription and translation, the expressed protein can diffuse and bind non-specifically to the slide around the printed spot. The “Halo ring” is produced when an AAb binds to the diffused protein. We compared spot intensity measurements of the same plasma sample tested on different two days to determine the consistency across experiments.

### Verification and Validation of plasma AAb biomarkers using ELISA

The 23 potential AAb biomarkers were analyzed with ELISA in duplicate as previously described 27. First, 96-well high-bind ELISA plates (Corning Inc, NY) were coated with goat anti-GST antibody (GE Healthcare, MA) at 10 μg/mL in 0.2 mol/L sodium bicarbonate buffer (pH 9.4) overnight at 4 °C. In parallel, the antigens were prepared by incubating cDNA plasmids (20 ng/mL) in a human cell-free expression system (Thermo Fisher, USA) for 2 hours at 30 ^o^C. The anti-GST antibody coated plate was incubated with 1:100 diluted expressed antigens, blocked with 5% milk in PBST at room temperature for 1 hour, and then incubated with 1:300 diluted plasma in PBST for 1 hour at room temperature. After washing the plate three times with PBST solution, AAb detection was performed by incubating the plate with an HRP-conjugated anti-human IgG antibody (Jackson ImmunoResearch Laboratories, PA, USA) for 1 hour, followed with tetramethylbenzidine (TMB) substrate (ComWin Biotech, Beijing, China) for 15 minutes at room temperature. The reaction was stopped with 2 M sulfuric acid. The signal was read using a SPETRA MAX190 plate reader (Molecular Devices, Sunnyvale, CA, USA) at 450 nm. The ELISA signal of each AAb was normalized using the OD450 of the expressed antigens divided by the median OD450 of all antigens measured for that sample as previously described 27.

HRP-conjugated anti-human IgG1-4 antibodies (Jackson ImmunoResearch Laboratories, PA, USA) were employed to detect the distribution of PD1 and PD-L1 AAb IgG subtypes in plasma.

### Statistical analysis

The Mann-Whitney *U* test was used to identify significantly-produced AAbs between the responder and non-responder groups among the ASPS, NSCLC and lymphoma patients (p≤0.05). The association between the AAbs and their predictive effect in determining whether a patient would be a responder or non-responder in ASPS, NSCLC and lymphoma patients was performed with a Spearman correlation coefficient (plotted with Circos 28). The tests were implemented by the Python Scipy Statsmodels module and plotted by the Python Matplotlib and Seaborn modules. Hierarchical clustering and Principle Component Analysis (PCA) were implemented and plotted by the statistical language R.

The discriminatory capacity of the selected biomarkers was assessed by the area under the curve (AUC) using GraphPad Prism version 8.0.1 for Windows (GraphPad Software, San Diego, California USA). To determine the performance of the PD1 and PD-L1 IgG2 AAbs, we used the Python Scikit-learn module to calculate the partial area under the receiver operating characteristic curve (pAUC) as well as the sensitivity and specificity using the filtered threshold (with a sensitivity > 0.1 and pAUC > 0.005) 29.

## Results

### Global identification of AAbs related to anti-PD1 therapy using NAPPA

The overall experimental design of this study is shown in Figure 1. We performed the AAb screening using NAPPA protein microarrays with plasma from ASPS, NSCLC and lymphoma patients (Figure S1A). Printing was evaluated using a fluorescent stain that binds to double-stranded DNA (i.e., PicoGreen), whereas *in vitro* protein expression was assessed by staining with a mouse anti-GST tag antibody and then a fluorescently-conjugated anti-mouse antibody (Figure S1D). 95% of the cDNA plasmids were successfully printed and the captured GST-tagged proteins were displayed with high-reproducibility (R=0.907) (Figure S1E). The reproducibility of plasma AAb detection using different NAPPA arrays was 0.95 (Figure S2).

In the biomarker discovery stage, 10 plasma samples from 5 responders and 5 non-responders in discovery cohort 1 were first screened using NAPPA microarrays displaying ~2,300 human proteins. Seven AAbs (GEMIN2, DDX49, EIF4E2, CCDC130, MRPL44, P53, FATE1) were identified in the responder group. Since no similar study has been performed previously and the data from discovery cohort 1 showed promising results, we chose to expand the screening for discovery cohort 2 to ~ 4,600 human proteins, which represented different proteins than the first ~2,300 protein array set. The discovery cohort 2 contained 35 plasma samples (12 ASPS, 13 NSCLC, 10 lymphoma) from 18 responders and 17 non-responders. Fourteen AAbs (RCN3, VMAC, PHACTR1, EIF3H, LPCAT4, UBALD1, ARFGAP1, CPLX2, ZNF280B, SIX2, TECA3, JUN, SPAG8, SIX3) were selected for further evaluation (Table S1). These AAbs were either differentially-produced between responders and non-responders based on our array data or their antigens were shown to have an association with cancer in previous studies.

### Verification of selected AAbs using ELISA

Using ELISA, the production of 23 AAbs in 74 patients in verification cohort prior to anti-PD1 therapy (12 ASPS, 16 NSCLC, 46 lymphoma) was measured (Table 1). These 23 AAbs included the 21 AAbs identified in initial discovery stage using NAPPA arrays, as well as PD1 AAb and PD-L1 AAb due to their functional relevance to cancer immunity and anti-PD1 therapy 30, 31. Notably, PD1 and PD-L1 AAbs were not included on the NAPPA arrays during the biomarker discovery phase of this study. To ensure that the ELISA results were reproducible, we compared data from the same plasma samples analyzed on the same day and across different days. The correlation was 0.99 and 0.96 within and across different experiments, respectively (Figure S3A-B). The intra- and inter-coefficient of variation (CV) were 3% and 7%, respectively (Figure S3C-D). These results demonstrate that our ELISA data are reliable and reproducible.

**Figure 2:**
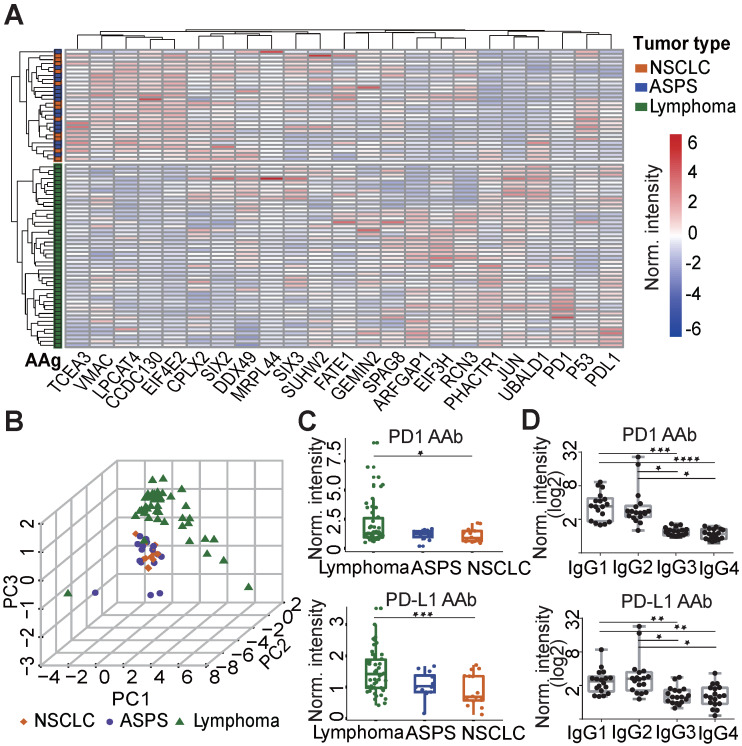
** Verification of AAbs using ELISA.** (A) Nonbiased hierarchical analysis of AAbs from ASPS, NSCLC and lymphoma patients prior to the anti-PD1 therapy. False-colored rainbow coloring from blue to red corresponds to the AAb level from low to high, respectively. The heat map and hierarchical cluster analysis data were normalized using the z-score. (B) Principal component analysis of AAbs in ASPS, NSCLC and lymphoma patients; (C) Detection of PD1 and PD-L1 AAbs in ASPS, NSCLC and lymphoma patients; (D) Detection of the IgG subtypes for PD1 and PD-L1 AAb subtypes using plasma from lymphoma patients. The data was log2 normalized by using the ELISA signal divided by the buffer background. The statistical analysis was performed using a Mann-Whitney *U* test with a p-value < 0.05. *, **, ***, **** in the graphs correspond to the p-value < 0.05, 0.01, 0.001 and 0.0001, respectively. ASPS: alveolar soft part sarcoma, NSCLC: non-small-cell lung cancer.

To elucidate the relationship between plasma AAbs and the different cancers explored in this study, hierarchical clustering was performed. The plasma AAb profile in lymphoma patients was distinct compared to ASPS and NSCLC patients. However, the AAb patterns between ASPS and NSCLC patients were similar (Figure 2A). Principle component analysis confirmed the clustering, in which almost all lymphoma patients were separated from ASPS and NSCLC patients (Figure 2B). Using the Mann-Whitney* U* test, we identified 7 AAbs (PD1, PD-L1, PHACTR1, ARFGAP1, EIF3H, JUN, UBALD1) that were significantly elevated in the lymphoma group, whereas 7 AAbs (EIF4E2, CCDC130, LPCAT4, CPLX2, SIX2, TCEA3, VMAC, MRPL44) were decreased in lymphoma patients than the other two groups (p≤0.05, Figure 2C, Figure S4). Notably, PD1 and PD-L1 AAbs were detected in a large number of cancer patients prior to anti-PD1 therapy, and the highest level was detected in lymphoma patients. Furthermore, IgG1 and IgG2 were the major IgG subtypes for both PD1 and PD-L1 AAbs (Figure 2D).

### Predictive AAb biomarkers of anti-PD1 therapy in ASPS patients

**Figure 3:**
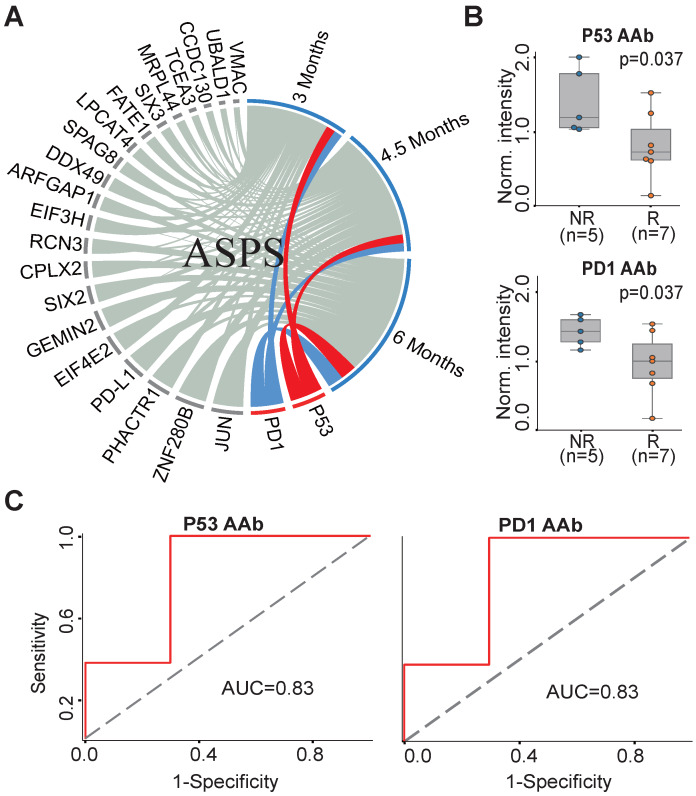
** AAb predictive biomarkers of anti-PD1 therapy response in ASPS patients.** (A) Association analysis of plasma AAbs and ASPS clinical response using circos association analysis at 3 months, 4.5 months, and 6 months, respectively. The association coefficient denotes the areas of the arc in the circle. Lines in red and blue represent the two most relevant markers with immunotherapy efficacy; (B) Box plot analysis of AAbs in ASPS responder and non-responder groups at 6 months. The statistical analysis was performed using a Mann-Whitney *U* test with a p-value ≤ 0.05; (C) Discrimination of ASPS patients' responses to anti-PD1 therapy by the P53 or PD1 AAbs using ROC curve analysis. R and NR represent the responder and non-responder groups, respectively. ASPS: alveolar soft part sarcoma.

To understand the association between the candidate 23 AAb biomarkers and the response to anti-PD1 therapy in ASPS patients, we analyzed the association of the baseline AAbs with ASPS patient response at 3 months, 4.5 months and 6 months. P53 and PD1 AAbs were well correlated with the responses of anti-PD1 therapy at all three time points (Figure 3A). The Mann-Whitney *U* test revealed that the P53 and PD1 AAbs were differentially produced between responders (n=7) and non-responders (n=5) at 6 months (p=0.037) (Figure 3B). The predictive performances of P53 and PD1 AAbs were evaluated using ROC curve analysis, which gave an AUC of 0.83 for both targets (Figure 3C).

### Predictive AAb biomarker of anti-PD1 therapy in NSCLC patients

**Figure 4:**
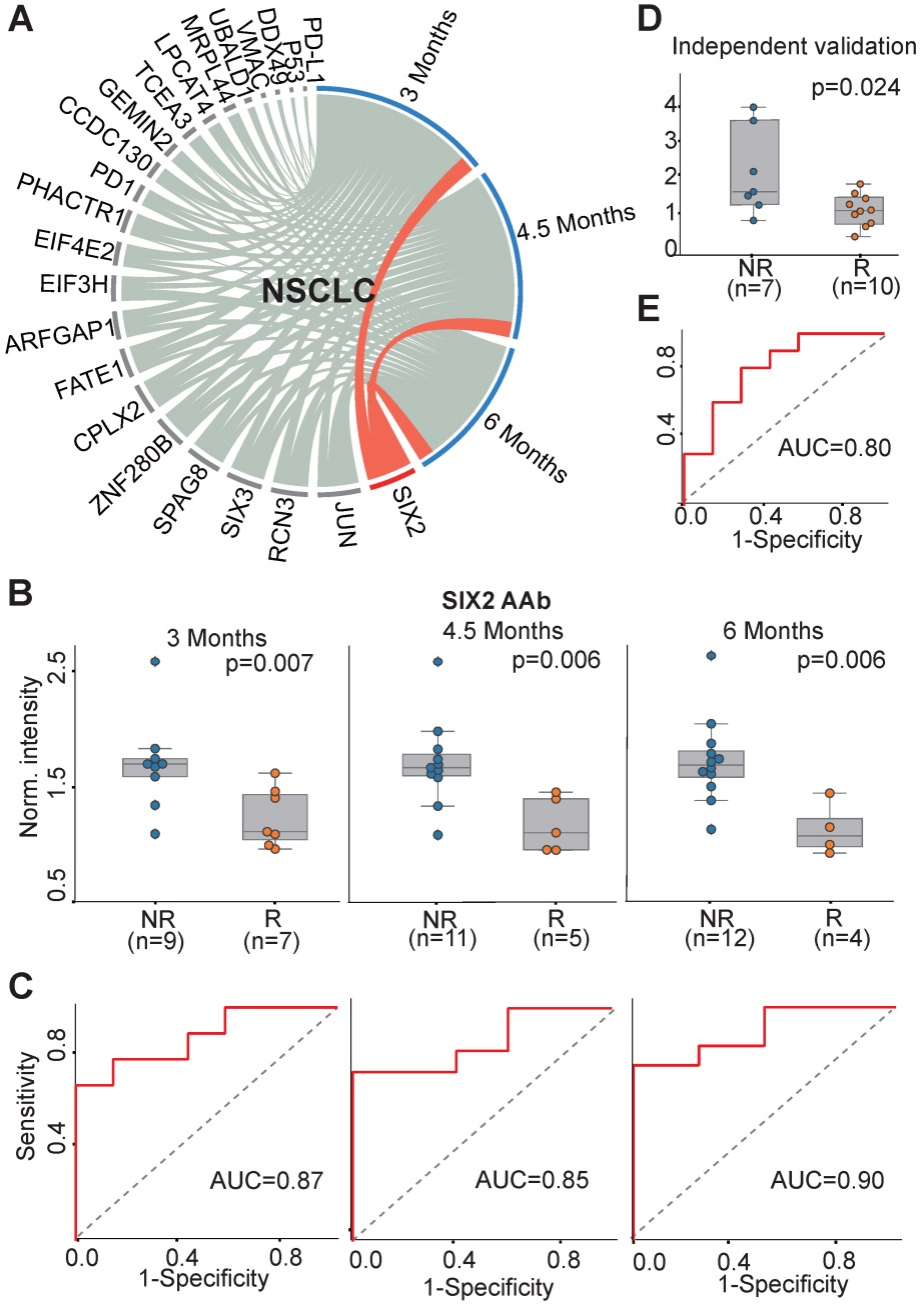
** AAb predictive biomarker of anti-PD1 therapy response in NSCLC patients.** (A) Circos correlation analysis of plasma AAbs and clinical response in NSCLC patients. The red line denotes the significant association between an AAb and response to anti-PD1 therapy in NSCLC patients. The correlation analysis was plotted with Circos; (B) Box plot analysis of the SIX2 AAb in the NSCLC responder and non-responder groups at 3 months, 4.5 months and 6 months. The statistical analysis was performed using a Mann-Whitney *U* test with a p-value ≤ 0.05; (C) Discrimination of NSCLC patients' responses to anti-PD1 therapy by the SIX2 AAb using ROC curve analysis. (D) and (E) Validation of SIX2 AAb in an independent group of NSCLC patients by box plot and ROC analysis, respectively. The statistical analysis was performed using a Mann-Whitney *U* test with a p-value ≤ 0.05; R and NR represent the responder and non-responder groups, respectively. NSCLC: non-small-cell lung cancer.

We investigated the association of all 23 AAbs with anti-PD1 therapy response in NSCLC patients. The concentration of SIX2 AAb before therapy was well correlated with the clinical outcome at all three evaluation time points (3 months, 4.5 months, 6 months) (Figure 4A). The SIX2 AAb level was significantly higher in non-responders than responders with p-values of 0.007, 0.006, 0.006 and AUCs of 0.87, 0.85 and 0.90 at 3 months, 4.5 months and 6 months, respectively (Figure 4B-C). In addition, we validated the SIX2 AAb in an independent cohort of NSCLC patients (n=17). The result indicated that the SIX2 AAb was consistently up-regulated in the non-responder group (p=0.024, Figure 4D) with an AUC of 0.80 at 3 months (Figure 4E).

### Predictive AAb biomarker of anti-PD1 therapy in lymphoma patients

**Figure 5:**
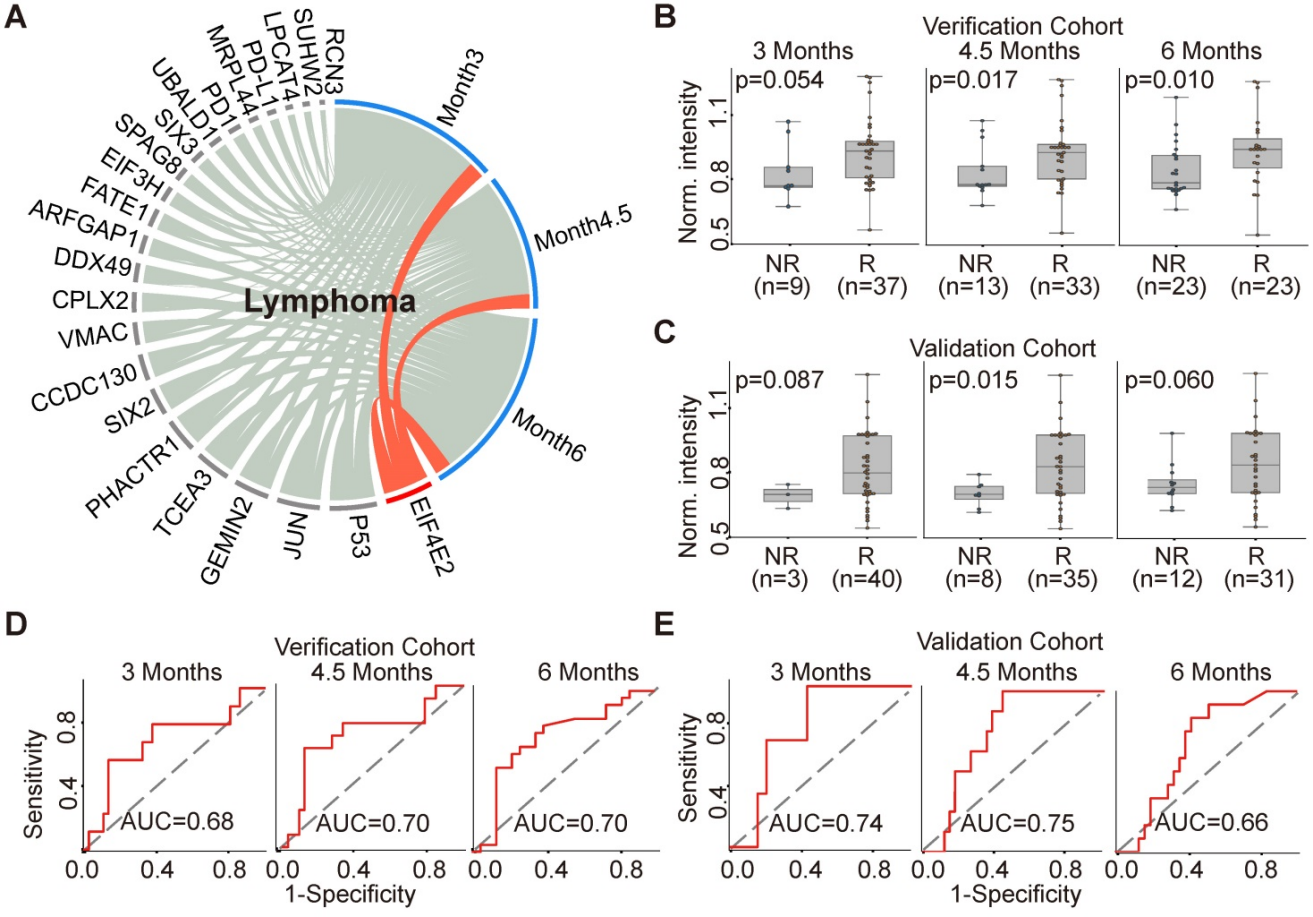
** AAb predictive biomarker of anti-PD1 therapy response in lymphoma patients.** (A) Circos correlation analysis of plasma AAbs and clinical response in lymphoma patients. The red line denotes the significant association between the AAb and response to anti- PD1 therapy in lymphoma patients. The correlation analysis was plotted with Circos; (B) and (C) Box plot analysis of the EIF4E2 AAb in the lymphoma responder and non-responder groups at 3 months, 4.5 months and 6 months in verification cohort and validation cohort, respectively; (D) and (E) Discrimination of lymphoma patients' responses to anti-PD1 therapy by the EIF4E2 AAb using ROC curve analysis in the verification and validation cohorts, respectively; The statistical analysis was performed using a Mann-Whitney *U* test with a p-value≤0.05, R and NR represent the responder and non-responder groups, respectively.

The production of EIF4E2 AAb correlated with clinical outcome in 46 lymphoma patients (Figure 5A). More specifically, the baseline EIF4E2 AAb was up-regulated in the responder group compared to non-responder group at 3 months, 4.5 months and 6 months with p-values of 0.054, 0.017, and 0.010, respectively (Figure 5B). The AUCs of EIF4E2 AAb for the evaluation time point of 3 months, 4.5 months and 6 months were 0.68, 0.70 and 0.70, respectively (Figure 5D). We further validated the EIF4E2 AAb in an independent cohort of 43 lymphoma patients, with p-values of 0.087, 0.015, and 0.060 and AUCs of 0.74, 0.75 and 0.66 for the evaluation time points at 3 months, 4.5 months and 6 months, respectively (Figure 5C,5E).

**Figure 6:**
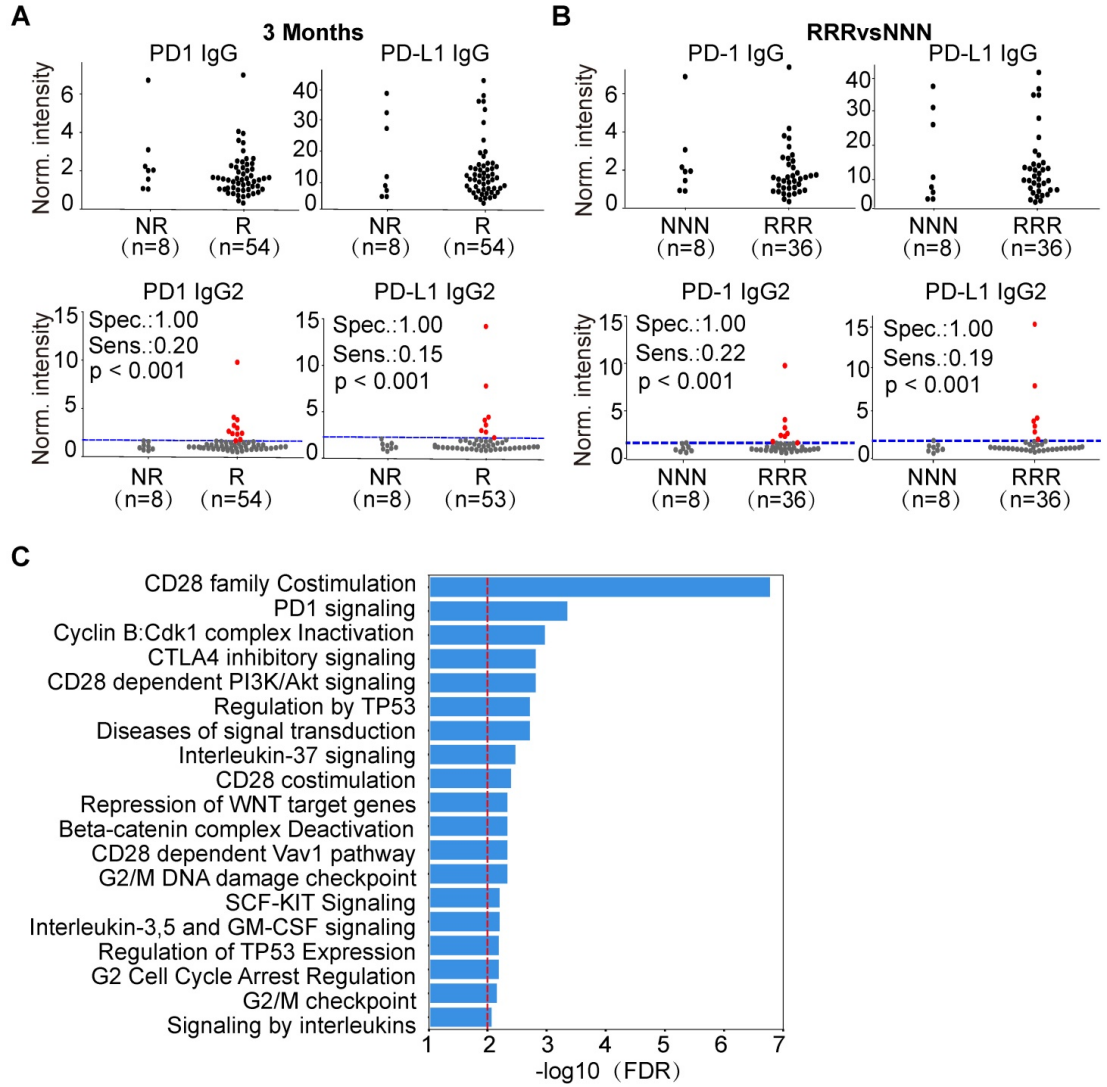
** Predictive potential of PD1,PDL1 IgG2 in lymphoma and bioinformatics analysis of AAb biomarkers.** (A) Jitter plot analysis of PD1 and PD-L1 IgG and IgG2 AAbs in the responder and non-responder groups of lymphoma patients at 3 months; (B) Jitter plot analysis of PD1 and PD-L1 IgG and IgG2 AAbs in lymphoma patients that were consistently responders or non-responders across all time points; (C) Pathway enrichment analysis of the antigens targeted by the AAb biomarkers and their protein interactions using the Reactome database. The red line represents an FDR ≤ 0.01; The RRR and NNN are defined as the patients that showed consistent responses and non-responses to anti-PD1 therapy at 3 months, 4.5 months and 6 months, respectively. The sensitivity and specificity were calculated by pAUC analysis. The p-values was calculated by Kolmogorov-Smirnov test via Python SciPy module. The patients with PD1 and PD-L1 IgG2 AAbs above the cut-off are shown as red dots.

We compared the levels of PD1 and PD-L1 IgG1 and IgG2 AAbs between responders and non-responders in ASPS, NSCLC and lymphoma. Interestingly, the production of PD1 and PD-L1 IgG2 AAbs was highly specific for a subset of lymphoma patients who responded to therapy (Figure 6A, Figure S5-S6), which may due to the various AAb distribution of IgG2 in lymphoma patients (Figure S7). Using pAUC analysis, the sensitivity and specificity of PD1 and PD-L1 IgG2 AAbs were determined. The sensitivity of PD1 IgG2 AAb was 21% (11/53), 21% (10/47), and 14% (5/37) at the evaluation time points of 3 months, 4.5 months and 6 months, respectively. The specificity of PD1 IgG2 AAb was 100% (9/9), 93% (14/15) and 92% (23/25) at 3 months, 4.5 months and 6 months, respectively. The sensitivity of PD-L1 IgG2 AAb was 15% (8/53), 28% (13/47) and 23% (9/37), whereas the specificity was 100% (9/9), 93% (14/15) and 92% (23/25) at 3 months, 4.5 months and 6 months, respectively (Figure 6A, Figure S5-S6, Table S2). These results were also observed in lymphoma patients who showed a consistent response or non-response to anti-PD1 therapy at all three evaluation time points. PD1 and PD-L1 IgG2 AAbs had a sensitivity of 22% (8/36) and 19% (7/36), respectively, at a specificity of 100% (8/8) in lymphoma patients who responded to anti-PD1 therapy (Figure 6B).

### Bioinformatics analysis of AAb biomarkers associated with anti-PD1 therapy

Using the random walking with restart (RWR) approach, 70 proteins (Figure S8) were identified that interact with the antigens targeted by the 5 AAb biomarkers (PD1, PD-L1, P53, SIX2, EIF4E2) from our study (Table 2, Table S3). Pathway enrichment analysis indicates that these proteins belong to pathways involved in cancer, immune checkpoint, and gene transcription and translation (Figure 6C, Table S4).

## Discussion

A variety of biomarkers have been developed to identify cancer patients who would benefit from anti-PD1 therapy. However, the clinical utility of these biomarkers has been limited for various reasons, including inconsistent methods, invasive or difficult-to-obtain samples (e.g., tumor DNA, tissue, circulating tumor cells), and varying clinical thresholds across different cancers 3. Thus, there is an urgent need for the identification of novel biomarkers that can predict immunotherapy response. In this study, we identified new predictive candidate AAb biomarkers of anti-PD1 therapy using protein microarrays. We then verified 5 AAb biomarkers with ELISA technology. To our knowledge, this is the first study investigating the association between AAbs and anti-PD1 therapeutic response by proteomics screening.

We identified distinct AAbs between ASPS, NSCLC and lymphoma patients. The different cell types in which the cancers originate may explain these data. ASPS and NSCLC arises from epithelial or mesenchymal cells, whereas lymphoma begins in lymphocytes of the immune system 32. We also found that the production of PD-L1 AAb was significantly higher in lymphoma patients than ASPS and NSCLC patients. Notably, PD-L1 is up-regulated in >95% of cases in HL 33.

Five potential AAb biomarkers (PD1, PD-L1, P53, SIX2, EIF4E2) predictive of patient response to anti-PD1 therapy were identified. PD1 and PD-L1 are immune checkpoint proteins that are critical in regulating antitumor immune response. The PD-L1 AAb has been observed in the plasma of patients with rheumatoid arthritis 34, type 1 autoimmune hepatitis 35, and systemic lupus erythematosus 36. However, the association of PD1 and PD-L1 AAbs with anti-PD1 therapy is unknown. In this study, we show that PD1 and PD-L1 AAbs are present in cancer patients. Furthermore, we demonstrate that the PD1 and PD-L1 AAbs are mainly comprised of the IgG1 and IgG2 subtypes in lymphoma. Importantly, our results indicate that in lymphoma patients, PD1 and PD-L1 IgG2 AAbs are highly produced in ~20% patients who respond to anti-PD1 therapy (Figure 6A, Figure S5-S7). Given these results, we propose that these AAbs may play important roles in anti-PD1 or anti-PD-L1 therapy. The molecular mechanisms of these biomarkers in ICB therapy should be elucidated in future studies.

P53 is the most well-known tumor suppressor gene, and the AAb to P53 has been found in different cancers including breast cancer, lung cancer, and ovarian cancer 37. Recent evidence indicated that the expression of PD1 and PD-L1 can be regulated by P53 in cancer cells with genotoxic stress and DNA damage 38. The expression of P53 also correlated with the malignancy and proliferation of ASPS tumors 39. In our study, both PD1 and P53 AAbs were significantly up-regulated in the non-responder group compared to responder group in ASPS patients (Figure 3B), thus supporting earlier studies 39.

SIX2 is a transcription factor that participates in signaling pathways associated with cell proliferation, differentiation, and survival 40. The mRNA expression of SIX2 is higher in tumors than normal tissues, and positively correlated with advanced stages and poorer survival of NSCLC patients 41. However, the immunogenicity of SIX2 is unknown. We observed and validated this negative association of SIX2 and clinical outcome in our NSCLC cohort (Figure 4).

The EIF4E2 AAb was consistently up-regulated in the responder group compared to non-responder group in lymphoma patients. Eukaryotic Translation Initiation Factor 4E Family Member 2 (EIF4E2) is a member of the cap-binding subunit (EIF4E) and part of the EIF4F complex. EIF4E plays a vital role in cap-dependent translation of cellular mRNAs, and influences a subset of mRNAs encoding oncogenic proteins, such as vascular endothelial growth factor (VEGF), MYC, and cyclins 42. EIF4E is frequently overexpressed in various types of cancers, and its overall phosphorylation is significantly higher in tumor tissue compared to paired normal tissues, thus supporting the critical role of EIF4E in cancer 43. Recent work indicated that EIF4F affects translation of the signal transducer and activator of transcription 1 (STAT1) protein, which leads to the overexpression of PD-L1 on the surface of cancer cells. They also observed a correlation between EIF4F complex activation and immunotherapy in melanoma patients 44. The overproduction of EIF4E2 AAb in lymphoma patients may inhibit the downstream translation of oncogenic proteins like VEGF, MYC, cyclins, and STAT1.

The current approved or promising predictive markers of anti-PD1 therapy require an invasive tissue biopsy, the results can be influenced by the heterogeneity of tumor tissue 29. In comparison, AAb detection using ELISA requires only a few microliters of plasma or serum. ELISA testing is also simple, affordable, rapid and can be completed within a few hours without sophisticated equipment or in-depth training. Serological AAb detection has been demonstrated to be a powerful approach for early cancer detection and efficacy monitoring (e.g., EarlyCDT-lung to test for lung cancer, Videssa® Breast to test for breast cancer)^45^. The results of this study further indicate that AAbs have clinical value to predict response of anti-PD1 therapy. Besides ASPS, NSCLC and lymphoma, the predictive value of these AAbs can be further investigated in other types of cancer. The functional association between these AAbs and immune checkpoint pathways has been illustrated by bioinformatics analysis (Figure 6C, Figure S8). However, further study is required to investigate the interactions of cancer cells and host B-cell immunity, and to evaluate the contribution of AAbs to cancer cell eradication.

There are several limitations in this study. Due to the low incidence of ASPS, there were a limited number of ASPS plasma samples in this study. The number of responders and non-responders differed across cohorts, which could be caused by various response rates across different cancer types. In addition, improved patient stratification would benefit from studying the longitudinal change of these AAb biomarkers pre- and post- anti-PD1 therapy. The predictive performance of the AAbs in combination with other biomarkers (e.g, PD-L1 expression) should also be explored.

In conclusion, this is the first study to systematically investigate the relationship between AAbs and the anti-PD1 therapeutic response using a high-throughput proteomics technology. The results suggest that AAbs can be a new class of predictive biomarkers for anti-PD1 therapy.

## Supplementary Material

Supplementary figures and tables.

## Figures and Tables

**Table 1:** Patient baseline characteristics

		Discovery cohort 1			Discovery cohort 2			Verification cohort			Validation cohort	
Characteristics		ASPS n=3(%)	NSCLC n=3(%)	Lymphoma n=4(%)	ASPS n=12(%)	NSCLC n=13(%)	Lymphoma n=10 (%)	ASPS n=12(%)	NSCLC n=16(%)	Lymphoma n=46(%)	NSCLC n=17(%)	Lymphoma n=43(%)
Age (year)												
	median	34	60	31	33	60	31	33	60.5	34	63	34
	range	33-41	43-62	28-53	22-48	32-67	22-53	22-48	32-74	18-69	33-81	21-60
Gender												
	Male	3(100)	3(100)	2(50)	5(42)	11(85)	5(50)	5(42)	14(88)	52(58)	12(71)	26(60)
	Female	0(0)	0(0)	2(50)	7(58)	2(15)	5(50)	7(58)	2(12)	37(42)	5(29)	17(20)
ECOG performance												
	0	1(33)	1(33)	3(75)	4(33)	2(15)	7 (70)	4(33)	4(25)	45(51)	17(100)	23(53)
	1	2(67)	2(67)	1(25)	8(67)	11(85)	3(30)	8(67)	12(75)	44(49)	0(0)	20(47)
Stage												
	I	0(0)	0(0)	0(0)	0(0)	0(0)	0(0)	0(0)	0(0)	2(2)	0(0)	1(2)
	II	0(0)	1(33)	2(50)	0(0)	1(8)	2(20)	0(0)	1(6)	16(18)	0(0)	12(28)
	III	0(0)	1(33)	1(25)	0(0)	3(23)	2(20)	0(0)	3(19)	9(10)	5(29)	24(56)
	IV	3(100)	1(33)	1(25)	12(100)	9(69)	6(60)	12(100)	12(75)	61(69)	11(65)	6(14)
	Unknown	0(0)	0(0)	0(0)	0(0)	0(0)	0(0)	0(0)	0(0)	1(1)	1 (6)	0
Clinical Benefit												
3 months												
	Responder	1(33)	1(33)	3(75)	9(75)	5(38)	9(90)	9(75)	7(44)	77(87)	10(59)	3(7)
	Non-responder	2(67)	2(67)	1(25)	3(25)	8(62)	1(10)	3(25)	9(56)	12(13)	7(41)	40(93)
4.5 months												
	Responder	1(33)	1(33)	3(75)	8(67)	4(31)	9(90)	8(67)	5(31)	68(76)		35(81)
	Non-responder	2(67)	2(67)	1(25)	4(33)	9(69)	1(10)	4(33)	11(69)	21(24)		8(19)
6 months												
	Responder	1(33)	1(33)	3(75)	7(58)	3(23)	8(80)	7(58)	4(25)	56(63)		31(72)
	Non-responder	2(67)	2(67)	1(25)	5(42)	10(67)	2(20)	5(42)	12(75)	33(37)		12(28)

ECOG: Eastern Cooperative Oncology Group

**Table 2:** Functional annotation of AAb biomarkers identified in this study

No.	Gene name	Protein name	UniProt ID	Protein class	Molecular function
1	TP53	Cellular tumor antigen p53	P04637	transcription factor (PC00218)	binding (GO:0005488); transcription regulator activity (GO:0140110)
2	PDCD1 (PD1)	Programmed cell death protein 1 (Protein PD1)	Q15116	-	apoptotic process, humoral immune response
3	CD274 (PD-L1)	Programmed cell death 1 ligand 1	Q9NZQ7	immunoglobulin receptor superfamily	cell surface receptor signaling pathway; negative regulation of activated T cell proliferation
4	SIX2	Homeobox protein SIX2	Q9NPC8	transcription factor (PC00218)	binding (GO:0005488); transcription regulator activity (GO:0140110)
5	EIF4E2	Eukaryotic translation initiation factor 4E type 2 (eIF-4E type 2)	O60573	translation initiation factor	binding (GO:0005488); transcription regulator activity (GO:0140110)
