# Supplementary material for: Autoantibody profiling identifies predictive biomarkers of response to anti-PD1 therapy in cancer patients
Source: Theranostics. 2020 May 16;10(14):6399–410. doi: 10.7150/thno.45816 (PMC7255026; doi:10.7150/thno.45816)
Supplement: Supplementary file 1 — Supplementary figures and tables. [file thnov10p6399s1.pdf]

Supplementary appendix

Autoantibody profiling identifies predictive biomarkers of response to anti-PD1 therapy in cancer patients

Supplemental Methods

Supplementary Tables: 4

Supplemental Figures: 8

Supplemental Tables：4

Supplemental Table S1	Autoantibodies selected by protein microarray fluorescent signal and prior knowledge.
Supplemental Table S2	Performance of PD1 IgG2 and PD-L1 IgG2 as predictive markers.
Supplemental Table S3	The human proteins associated with five AAb biomarkers identified by the random walking with restart (RWR) approach.
Supplemental Table S4	Pathway enrichment analysis of the AAb biomarkers and their protein interactions using the Reactome database.

Supplemental Figures: 8

Supplementary	Schematic illustration of protein microarray preparation and

Figure S1	plasma AAb screening.
Supplementary Figure S2	Reproducibility of plasma autoantibody detection using NAPPA protein microarrays.
Supplementary Figure S3	Reproducibility of serological antibody detection using ELISA.
Supplementary Figure S4	Jitter plot analysis of differentially expressed plasma AAbs in ASPS, NSCLC and lymphoma patients.
Supplementary Figure S5	Comparison of PD1 and PD-L1 AAb expression between the responder and non-responder lymphoma patient groups at the evaluation time point of 4.5 months.
Supplementary Figure S6	Comparison of PD1 and PD-L1 AAb expression between the responder and non-responder lymphoma patient groups at the evaluation time point of 6 months.
Supplementary Figure S7	Distribution of PD1/PD-L1 IgG and IgG2 AAb expression in consistent responder and non-responder lymphoma patients.
Supplementary Figure S8	Protein-protein interaction network of the AAb biomarkers.

Supplementary Methods

Bioinformatics analysis

The circus correlation analysis was performed using software at http://www.circos.ca/ and plotted with Circos[1]. The protein class analysis for the proteins displayed on the NAPPA array, candidate markers and PPI subnetwork were performed using the PANTHER database[2]. Prior evidence of the candidate markers were derived from the human AAg database AAgAtlas (http://biokb.ncpsb.org/aagatlas/)[3]. The human protein-protein interaction subnetwork analysis was performed using the IntAct database[4] by random walking[5]. The pathway enrichment analysis was performed using the Reactome database [6].

Functional analysis of validated AAb biomarkers by protein-protein interactions

Five protein antigens (PD1, PD-L1, P53, SIX2, EIF4E2) of the identified AAb biomarkers in this study were selected together as seed nodes. The random walking with restart (RWR) approach was then employed to prioritize the relativity of the other human proteins compared to the five markers with the steadily reaching probability at the convergence state[5]. For the subnetwork construction, the largest average clustering coefficient of the subnetwork composed of the higher prioritized proteins determined the threshold of the top-rank proteins. For RWR in the human protein-protein interaction network, each edge was weighted with the reciprocal of the given node's degree. The restarting parameter of RWR was set at 0.7 for simplicity[5]. The subnetwork analysis was implemented and plotted with the Python Networkx and Matplotlib modules, respectively.

References

1. Krzywinski M, Schein J, Birol I, Connors J, Gascoyne R, Horsman D, et al. Circos: an information aesthetic for comparative genomics. Genome Res. 2009; 19: 1639-45.
2. Mi H, Muruganujan A, Ebert D, Huang X, Thomas PD. PANTHER version 14: more genomes, a new PANTHER GO-slim and improvements in enrichment analysis tools. Nucleic Acids Res. 2019; 47: D419-D26.
3. Wang D, Yang L, Zhang P, LaBaer J, Hermjakob H, Li D, et al. AAgAtlas 1.0: a human autoantigen database. Nucleic Acids Res. 2017; 45: D769-D76.
4. Alonso-Lopez D, Campos-Laborie FJ, Gutierrez MA, Lambourne L, Calderwood MA, Vidal M, et al. APID database: redefining protein-protein interaction experimental evidences and binary interactomes. Database (Oxford). 2019; 2019.
5. Kohler S, Bauer S, Horn D, Robinson PN. Walking the interactome for prioritization of candidate disease genes. Am J Hum Genet. 2008; 82: 949-58.
6. Fabregat A, Jupe S, Matthews L, Sidiropoulos K, Gillespie M, Garapati P, et al. The Reactome Pathway Knowledgebase. Nucleic Acids Res. 2018; 46: D649-D55.

7. Yu X, Wallstrom G, Magee DM, Qiu J, Mendoza DE, Wang J, et al. Quantifying antibody binding on protein microarrays using microarray nonlinear calibration. Biotechniques. 2013; 54: 257-64.
8. Wang H, Demirkan G, Bian X, Wallstrom G, Barker K, Karthikeyan K, et al. Identification of Antibody Against SNRPB, Small Nuclear Ribonucleoprotein-Associated Proteins B and B', as an Autoantibody Marker in Crohn's Disease using an Immunoproteomics Approach. J Crohns Colitis. 2017; 11: 848-56.

Supplementary Figures

Figure S1

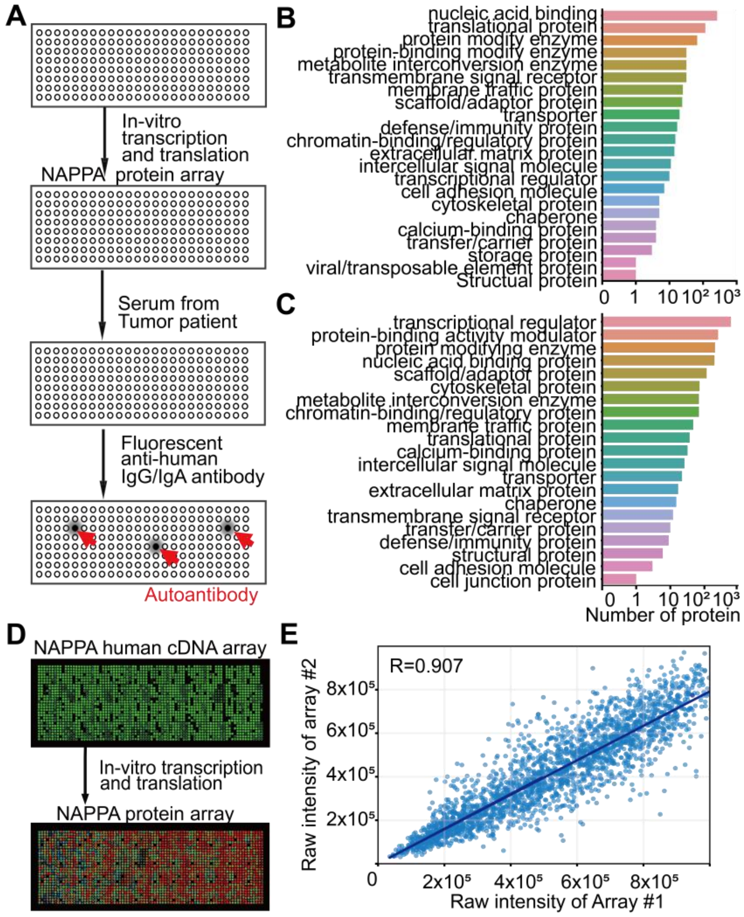

Figure S1. Schematic illustration of protein microarray preparation and plasma AAb screening. (A) Workflow of serum AAb detection using self-assembed protein microarrays; (B) Protein class analysis of 2300 human proteins used for screening of discovery cohort 1. (C) Protein class analysis of 4600 human proteins used for screening of discovery cohort 2; (D) Representative images of human cDNA microarray and protein microarrays; (E) Correlation between the fluorescent signals of different protein microarrays with anti-GST antibody staining representing levels of displayed proteins;

Figure S2

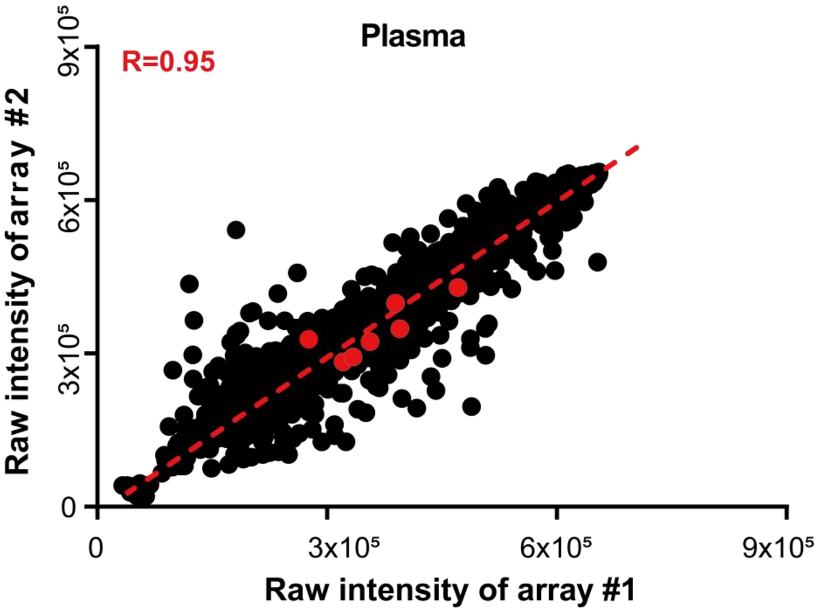

Figure S2. Reproducibility of plasma AAb detection using NAPPA protein microarrays. The autoantibody for the same plasma sample was detected using NAPPA protein microarray on different days. The red spots indicated positive controls.

Figure S3

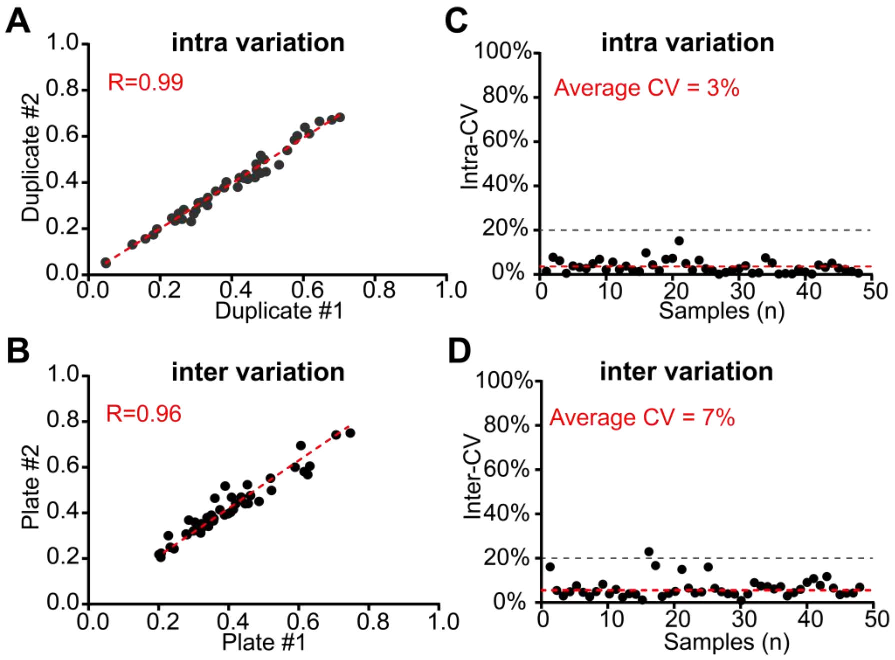

Figure S3. Reproducibility of serological antibody detection using ELISA. (A) and (B) are the correlation analyses of ELISA signals within and between different 96-well plates, respectively. (C) and (D) are the intra-CV and inter-CV of ELISA assays within and across different experiments as previously described [7].

Figure S4

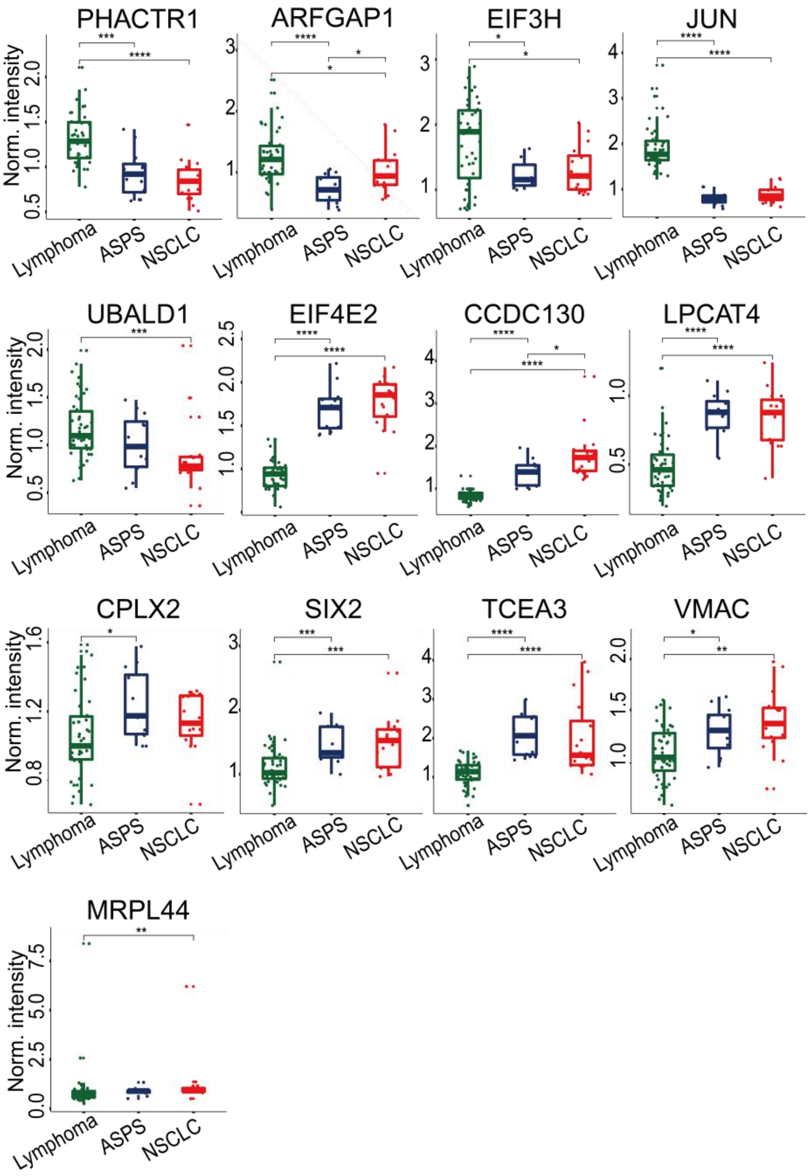

Figure S4. Jitter plot analysis of differentially-expressed plasma AAbs in ASPS, NSCLC and lymphoma patients. The statistical analysis was performed using the Mann-Whitney U test. *, **, ***, **** in the graphs correspond to a p-value of <0.05, <0.01, <0.001 and <0.0001, respectively.

Figure S5

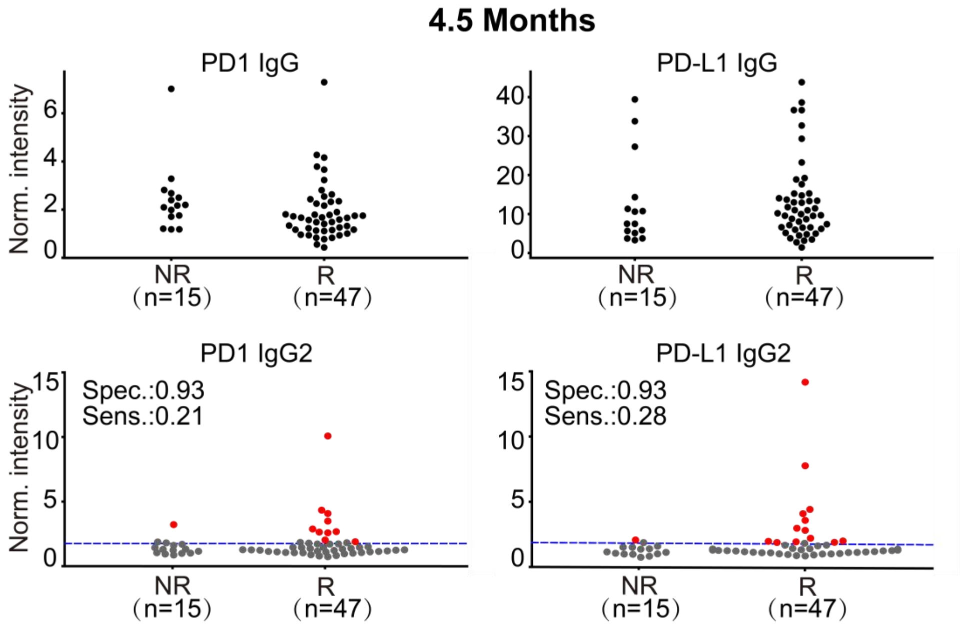

Figure S5. Comparison of PD1 and PD-L1 AAb expression between the responder and non-responder lymphoma patient groups at the evaluation time point of 4.5 months. Patients with PD1 IgG2 and PD-L1 IgG2 AAbs above the cut-off are shown as red dots.

Figure S6

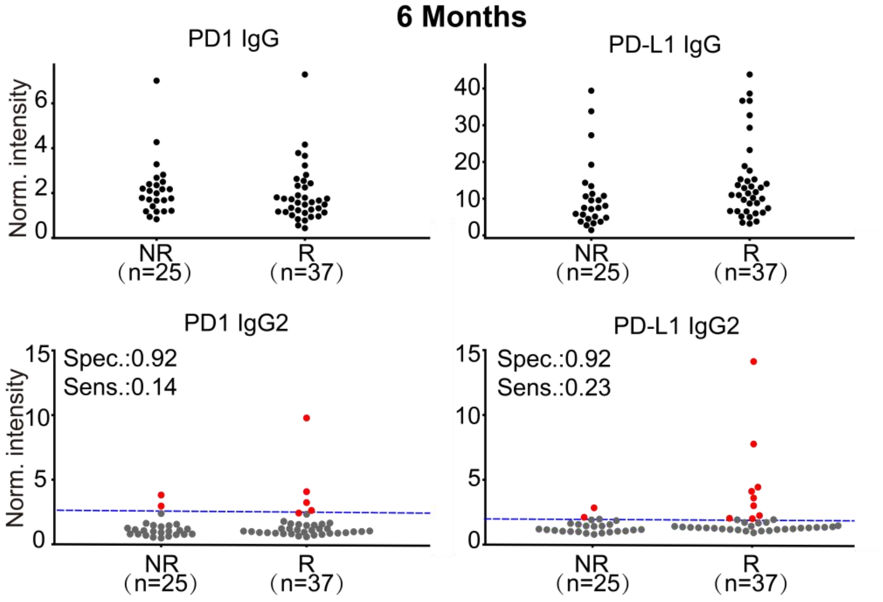

Figure S6. Comparison of PD1 and PD-L1 AAb expression between the responder and non-responder lymphoma patient groups at the evaluation time point of 6 months. Patients with PD1 IgG2 and PD-L1 IgG2 AAbs above the cut-off are shown as red dots.

Figure S7

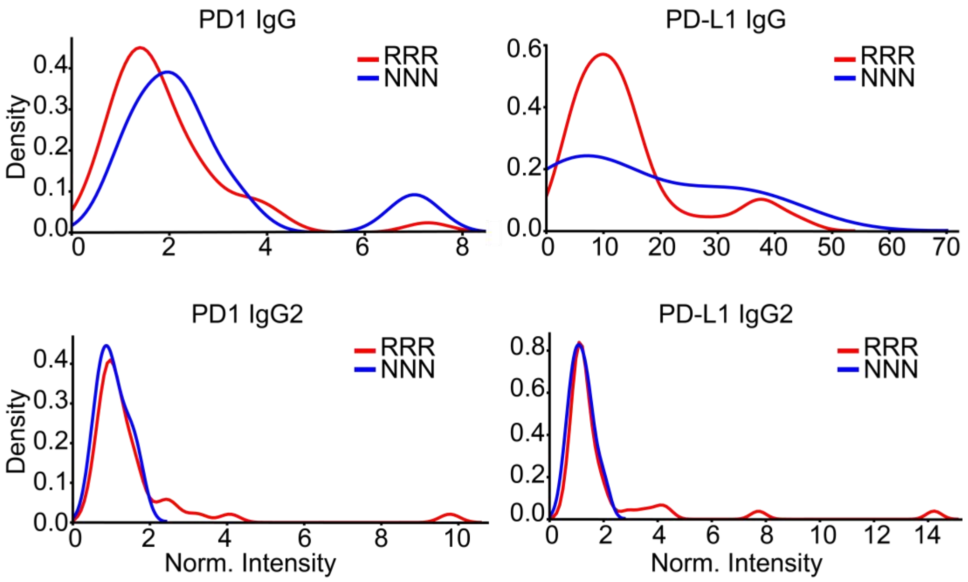

Figure S7. Distribution of PD1/PD-L1 IgG and IgG2 AAb expression in consistent responder and non-responder lymphoma patients. RRR and NNN are defined as patients that showed consistent response (R) and non-response (NR) to PD1 immunotherapy at 3 months, 4.5 months, and 6 months. The sample distribution of the PD1/PDL1 IgG and IgG2 values was based on the Gaussian kernel density estimation, which was implemented and plotted with the Python Seaborn module.

Figure S8

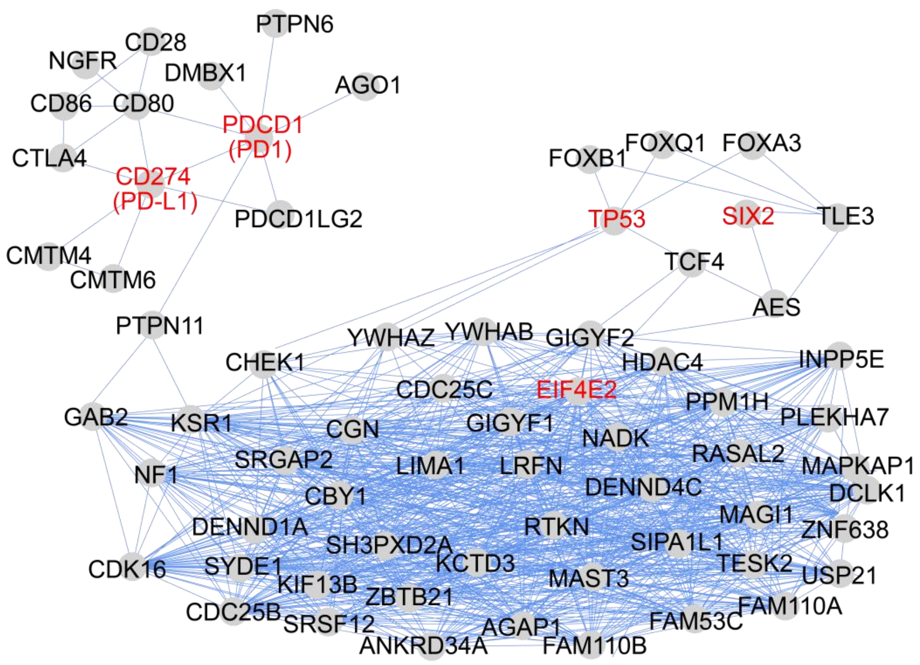

Figure S8. Protein-protein interaction network of the AAb biomarkers. The human protein-protein interaction subnetwork analysis of 5 picked markers (EIF4E2, P53, SIX2, PD-L1, PD1) was based on the protein-protein interaction database IntAct. The five markers were selected together as the seed nodes and the random walking with restart (RWR) approach was employed to prioritize the relativity of the other human proteins with the five markers with the steadily reaching probability at the convergence state. For the subnetwork construction, the threshold of the top-rank proteins was determined when the average clustering coefficient of the subnetwork composed of the higher prioritized proteins above the threshold was largest. For RWR in the human protein-protein interaction network, the edge from a given node to another node was weighted with the reciprocal of the given node's degree. The restarting parameter of RWR was set at 0.7 for simplicity. The subnetwork analysis

was implemented and plotted with the Python Networkx and Matplotlib modules, respectively.

Supplementary Table S1. Autoantibodies selected by protein microarray fluorescent signal and prior knowledge.

Ptient No.	1	2	3	4	5	6	7	8	9	10	11	12	13	14	15	16	17	18	19	20	21	22	23	24	25	26	27	28	29	30	31	32	33	34	35	36	Evidence in AAg Atlas database	Association with cancer（PMID）	
Cancer	L	L	L	LC	A	A	L	LC	L	LC	L	L	L	L	A	A	A	A	A	LC	LC	L	A	A	LC	LC	L	LC	LC	LC	LC	LC	LC	A	A	A			
Response-6M	R	R	R	R	R	R	R	R	R	R	R	R	R	R	R	R	R	R	R	NR	NR	NR	NR	NR	NR	NR	NR	NR	NR	NR	NR	NR	NR	NR	NR	NR			
**Discovery stage1**																																							
GEMIN2	0	0	0	0	5															0	0	0	0	0													Yes	Yes	29371219
DDX49	4	0	0	0	0															0	0	0	0	0													Yes	Yes	29618122
EIF4E2	0	5	0	0	0															0	0	0	0	0													Yes	Yes	24408918
CCDC130	0	0	0	0	5															0	0	0	0	0													Yes	Yes	22276133
MRPL44	0	0	0	5	0															0	0	0	0	0													Yes	Yes	25590838
P53	0	0	3	0	0															0	0	0	0	0													Yes	Yes	19410540
FATE1	0	0	0	4	0															0	0	0	0	0														Yes	31036566
**Discovery stage2**																																							
RCN3	0		0	0	0	0	3	0	0	0	0	3	3	0	0	0	0	0	0	0	0	0	0	0	0	0	0	0	0	0	0	0	0	0	0	0		Yes	27156316
VMAC	5		0	5	3	0	0	0	0	0	0	0	0	0	0	4	0	0	0	0	0	0	0	0	0	0	0	0	0	0	0	0	0	0	0	0		Yes	30248895
PHACTR1	0		0	0	0	0	0	0	0	0	0	0	0	0	0	0	0	0	0	0	0	4	0	0	0	0	0	0	0	0	5	0	0	0	0	0	Yes	Yes	23479725
EIF3H	0		0	0	0	0	0	0	0	0	0	0	0	0	0	0	0	0	0	0	0	0	0	0	0	0	0	0	3	3	0	0	0	0	0	0		Yes	25849773
LPCAT4	0		0	0	0	0	0	0	0	0	5	0	0	0	0	5	0	0	0	0	0	0	0	0	0	0	0	0	0	0	0	0	0	0	0	0		Yes	23815430
UBALD1	4		0	3	0	0	0	0	0	0	0	0	0	0	0	0	0	0	0	0	0	0	0	0	0	0	0	0	0	0	0	0	0	0	0	0	Yes	Yes	29416781
ARFGAP1	0		0	4	0	0	0	0	0	0	0	4	0	0	0	0	0	0	0	0	0	0	0	0	0	0	0	0	0	0	0	0	0	0	0	0	Yes	Yes	23752192
CPLX2	0		4	0	0	0	0	0	0	0	0	0	0	0	0	4	0	0	0	0	0	0	0	0	0	0	0	0	0	0	0	0	0	0	0	0	Yes	Yes	23912489
ZNF280B	0		0	0	5	0	0	0	0	0	0	0	0	0	0	0	4	0	0	0	0	0	0	0	0	0	0	0	0	0	0	0	0	0	0	0		Yes	22219177
SIX2	0		0	3	0	0	0	0	0	0	0	0	0	0	0	0	0	0	4	0	0	0	0	0	0	0	0	0	0	0	0	0	0	0	0	0		Yes	27821176
TCEA3	3		0	0	0	5	0	0	0	0	0	0	0	0	0	0	0	0	0	0	0	0	0	0	0	0	0	0	0	0	0	0	0	0	0	0		Yes	23357533
JUN	0		0	0	0	5	0	0	0	0	0	0	0	0	0	0	0	0	0	0	4	0	0	0	0	0	0	0	0	3	0	0	0	0	5	0	Yes	Yes	17057737

SPAG8	0	0	0	0	0	0	0	0	0	4	0	0	0	0	0	0	0	0	0	0	0	0	0	0	0	0	0	0	0	0	0	0	0	0	0	Yes	Yes	21150711
SIX3	0	0	0	0	4	0	0	0	0	5	0	4	0	0	0	0	0	0	0	0	5	0	0	0	0	0	0	0	0	0	0	0	0	0	0		Yes	27821176
PD1																																				Yes	Yes	22437870
PD-L1																																					Yes	22437870

Abbreviation: L:Lymphoma; LC:Lung cancer; A:Alveolar soft part sarcoma; 6M:6 Months; R:Responder; NR：Non-responder;

AAg Atlas database: http://biokb.ncpsb.org/aagatlas_portal/index.php

The numbers 1-5 indicate the signal intensity of “Halo ring”[8].

Supplementary Table S2. Performance of PD1 IgG2 and PD-L1 IgG2 as predictive markers.

Month	Feature	Threshold	Number<threshold	Number>threshold	Specificity	Sensitivity	pAUC
3months	PD1 IgG2	1.629	51	11	1.000	0.208	0.020
3months	PD-L1 IgG2	2.034	54	8	1.000	0.148	0.015
4.5months	PD1 IgG2	1.629	51	11	0.933	0.213	0.013
4.5months	PD-L1 IgG2	1.765	48	14	0.933	0.277	0.021
6months	PD1 IgG2	2.398	55	7	0.920	0.135	0.011
6months	PD-L1 IgG2	1.840	51	11	0.920	0.227	0.020
RRRvsNNN	PD1 IgG2	1.629	36	8	1.000	0.222	0.022
RRRvsNNN	PD-L1 IgG2	2.034	37	7	1.000	0.194	0.019

Supplementary Table S3. The human proteins associated with five AAb biomarkers identified by the random walking with restart (RWR) approach.

Ranking position	Gene symbol	Clustering coefficient
1	PDCD1	0.000
2	CD274	0.000
3	TP53	0.000
4	SIX2	0.000
5	EIF4E2	0.000
6	AES	0.000
7	TLE3	0.333
8	CD80	0.667
9	PDCD1LG2	0.630
10	CTLA4	0.617
11	CMTM6	0.542
12	CMTM4	0.661
13	DMBX1	0.585
14	PTPN6	0.533
15	PTPN11	0.493
16	AGO1	0.460
17	CD86	0.462
18	CD28	0.468
19	NGFR	0.437
20	KSR1	0.415
21	YWHAZ	0.395
22	GIGYF1	0.430
23	TCF4	0.423
24	CBY1	0.457
25	SRGAP2	0.491
26	KIF13B	0.516
27	ZBTB21	0.539
28	SH3PXD2A	0.559
29	LRFN1	0.578
30	DENND1A	0.596
31	HDAC4	0.610
32	KCTD3	0.625
33	LIMA1	0.639
34	SIPA1L1	0.651
35	MAST3	0.662
36	FOXB1	0.625
37	PPM1H	0.637
38	PLEKHA7	0.648
39	FOXQ1	0.627
40	RTKN	0.637
41	MAGI1	0.647
42	ZNF638	0.656
43	CHEK1	0.652
44	FOXA3	0.634
45	DCLK1	0.643
46	TESK2	0.652
47	DENND4C	0.660
48	CGN	0.666
49	NF1	0.674
50	SRSF12	0.681
51	YWHAB	0.676
52	SYDE1	0.683
53	MAPKAP1	0.689
54	GAB2	0.699
55	CDC25B	0.705
56	CDK16	0.710
57	AGAP1	0.716
58	INPP5E	0.716
59	CDC25C	0.717
60	NADK	0.723
61	FAM110A	0.723
62	GIGYF2	0.723
63	USP21	0.726
64	FAM53C	0.729
65	RASAL2	0.732
66	FAM110B	0.735
67	ANKRD34A	0.738
68	ANXA1	0.723
69	MAP3K21	0.727
70	GOLGA2	0.723
71	PHLDB2	0.726
72	TIAM1	0.730
73	CAMSAP2	0.732
74	KIF1C	0.734
75	KRT31	0.729

Supplementary Table S4. Pathway enrichment analysis of the AAb biomarkers and their protein interactions using the Reactome database.

Pathway identifier	Pathway name	#Entities found	#Entities total	Entities ratio	Entities pValue	Entities FDR	#Reactions found	#Reactions total	Reactions ratio	Species identifier	Species name
R-HSA-388841	Costimulation by the CD28 family	10	97	0.007	<0.001	<0.001	21	34	0.003	9606	Homo sapiens
R-HSA-75035	Chk1/Chk2(Cds1) mediated inactivation of Cyclin B:Cdk1 complex	4	15	0.001	<0.001	<0.001	4	5	<0.001	9606	Homo sapiens
R-HSA-389948	PD-1 signaling	5	45	0.003	<0.001	0.001	4	4	<0.001	9606	Homo sapiens
R-HSA-389513	CTLA4 inhibitory signaling	4	25	0.002	<0.001	0.002	4	5	<0.001	9606	Homo sapiens
R-HSA-389357	CD28 dependent PI3K/Akt signaling	4	26	0.002	<0.001	0.002	3	9	0.001	9606	Homo sapiens
R-HSA-3700989	Transcriptional Regulation by TP53	12	486	0.034	<0.001	0.002	174	259	0.021	9606	Homo sapiens
R-HSA-5663202	Diseases of signal transduction	12	489	0.035	<0.001	0.002	38	289	0.024	9606	Homo sapiens
R-HSA-9008059	Interleukin-37 signaling	4	36	0.003	<0.001	0.003	1	14	0.001	9606	Homo sapiens
R-HSA-389356	CD28 co-stimulation	4	39	0.003	<0.001	0.004	12	19	0.002	9606	Homo sapiens
R-HSA-4641265	Repression of WNT target genes	3	16	0.001	<0.001	0.005	7	7	0.001	9606	Homo sapiens
R-HSA-69473	G2/M DNA damage checkpoint	5	81	0.006	<0.001	0.005	7	12	0.001	9606	Homo sapiens
R-HSA-3769402	Deactivation of the beta-catenin transactivating complex	4	44	0.003	<0.001	0.005	11	14	0.001	9606	Homo sapiens
R-HSA-389359	CD28 dependent Vav1 pathway	3	17	0.001	<0.001	0.005	5	6	<0.001	9606	Homo sapiens
R-HSA-1433557	Signaling by SCF-KIT	4	50	0.004	<0.001	0.006	9	36	0.003	9606	Homo sapiens
R-HSA-512988	Interleukin-3, Interleukin-5 and GM-CSF signaling	4	50	0.004	<0.001	0.006	9	38	0.003	9606	Homo sapiens
R-HSA-6804754	Regulation of TP53 Expression	2	4	0.000	<0.001	0.006	5	5	<0.001	9606	Homo sapiens
R-HSA-6804114	TP53 Regulates Transcription of Genes Involved in G2 Cell Cycle Arrest	3	21	0.001	<0.001	0.006	6	11	0.001	9606	Homo sapiens
R-HSA-69481	G2/M Checkpoints	6	154	0.011	<0.001	0.007	12	24	0.002	9606	Homo sapiens
R-HSA-449147	Signaling by Interleukins	12	641	0.045	<0.001	0.009	21	492	0.041	9606	Homo sapiens
